# Radiation-induced alternative transcription and splicing events and their applicability to practical biodosimetry

**DOI:** 10.1038/srep19251

**Published:** 2016-01-14

**Authors:** Ellina Macaeva, Yvan Saeys, Kevin Tabury, Ann Janssen, Arlette Michaux, Mohammed A. Benotmane, Winnok H. De Vos, Sarah Baatout, Roel Quintens

**Affiliations:** 1 Radiobiology Unit, Belgian Nuclear Research Centre, SCK•CEN, Mol, Belgium; 2 Department of Molecular Biotechnology, Ghent University, Ghent, Belgium; 3 Data Mining and Modelling for Biomedicine Group, VIB Inflammation Research Center, Zwijnaarde, Belgium; 4 Department of Respiratory Medicine, Ghent University, Ghent, Belgium; 5 Department of Veterinary Sciences, University of Antwerp, Antwerp, Belgium

## Abstract

Accurate assessment of the individual exposure dose based on easily accessible samples (e.g. blood) immediately following a radiological accident is crucial. We aimed at developing a robust transcription-based signature for biodosimetry from human peripheral blood mononuclear cells irradiated with different doses of X-rays (0.1 and 1.0 Gy) at a dose rate of 0.26 Gy/min. Genome-wide radiation-induced changes in mRNA expression were evaluated at both gene and exon level. Using exon-specific qRT-PCR, we confirmed that several biomarker genes are alternatively spliced or transcribed after irradiation and that different exons of these genes exhibit significantly different levels of induction. Moreover, a significant number of radiation-responsive genes were found to be genomic neighbors. Using three different classification models we found that gene and exon signatures performed equally well on dose prediction, as long as more than 10 features are included. Together, our results highlight the necessity of evaluating gene expression at the level of single exons for radiation biodosimetry in particular and transcriptional biomarker research in general. This approach is especially advisable for practical gene expression-based biodosimetry, for which primer- or probe-based techniques would be the method of choice.

The recent nuclear accident at Fukushima Daiichi in March 2011 and the subsequent growing concerns about large-scale human radiation exposure have triggered the widespread recognition that there is an urgent need for effective biodosimetry tools that are capable of confirming or quantifying exposure to radiation in large cohorts of individuals potentially exposed to unknown doses for triage and personalized treatment^1^.

Following the Fukushima accident, the only individuals who received effective radiation doses of over 100 mSv, were 173 emergency and mitigation workers. Despite this generally low radiation exposure, which was clearly below the threshold of acute radiation disease, about 90,000 people were evacuated as a preventive safety action. This measure reduced the levels of possible exposure but also resulted in a number of evacuation-related deaths due to stress and/or lack of medical and social welfare facilities^2^. Hence, a rapid and accurate biodosimetry method would reduce the uncertainty about received doses and may mitigate psychological and health problems related to additional stress among the individuals who may or may have not been exposed to radiation (the so-called “worried wells”)^3^,^4^.

Cytogenetic measurements, more specifically dicentric assays, are considered the gold standard in biodosimetry^5^. While reliable and applicable to assess doses as low as 100 mGy, this method is time-consuming and laborious, and is not amenable to rapid diagnostics. A promising alternative technique consists in using gene expression data. Indeed, experimental data obtained by means of microarrays^6^, quantitative nuclease protection assay^7^, NanoString technology^8^, quantitative PCR (qPCR)^9^, or chemical ligation-dependent probe amplification (CLPA) assay^10^ have proven most efficient in accurately and rapidly assessing radiation exposure.

Several recent studies have shown that transcriptome analysis at the individual exon level may significantly add to our understanding of the transcriptional response to radiation exposure^11^,^12^,^13^. In particular, alternative transcription and alternative pre-mRNA splicing dramatically expand the translational repertoire. We hypothesise that alternative transcription and splicing analyses applied in the context of radiation exposure may generate additional radiation biomarkers with potentially increased sensitivity.

To test our hypothesis, we established gene and exon signatures that may serve as radiation biomarkers and subsequently compared their reliability and effectiveness. We opted for two X-ray doses relevant for triage purposes (0.1 and 1.0 Gy) and compared these to sham-irradiated control samples. We evaluated the predictive performance of gene and exon signatures using three different statistical models, which were further used to assess the robustness of our gene signature on an independent, publicly available dataset (Fig. 1). Our results yield new insights into transcriptional biomarker identification studies using genome-wide strategies and underline the importance of investigating gene expression at the single exon level.

## Results

### Low- and high-dose X-irradiation results in up-regulation of common genes

Whole-genome microarrays were used to analyse genome-wide transcriptional changes in human peripheral blood mononuclear cells (PBMCs) at 8 h after exposure to X-ray doses of 0.1 and 1.0 Gy compared to sham-irradiated control cells. Three-way ANOVA revealed 125 significantly differentially expressed genes (FDR < 0.05) between different doses of X-rays (Supplementary Table S1). Of these, the large majority (90.4%) were dose-dependently induced (Pearson’s correlation coefficients in Supplementary Table S1). Gene expression changes in response to radiation exposure were not gender-dependent (column FDR-corrected p-value (Dose*Gender) in Supplementary Table S1), as also previously suggested^14^. Unsupervised hierarchical clustering yielded a clear separation of the samples depending on the exposure dose (except for 3 out of 20 low dose-irradiated samples, which clustered together with the control samples) (Fig. 2a). Similar results were obtained using principal component analysis (PCA) of the same dataset (Fig. 2b). Comparison of the controls with each of the different doses yielded significantly increased expression levels following exposure to 0.1 Gy in 23 genes. Of these, 20 genes (87%) were also differentially expressed in cells irradiated with 1.0 Gy (Fig. 2c). Unsupervised hierarchical clustering using this subset of overlapping genes resulted in a perfect separation of the samples by exposure dose (Fig. 2d).

### X-irradiation induces alternative transcription and splicing

Considering the well-documented ability of ionising radiation to induce alternative gene splicing/transcription^11^,^15^, we first performed an Alternative Splicing ANOVA to identify which genes produced alternative transcripts following X-ray irradiation (Supplementary Table S2). Our results were in accordance with those of Sprung and co-authors^11^ for the most significant genes, despite differences in experimental models (e.g. PBMCs versus lymphoblastoid cell lines) and conditions (doses, time points and gene expression platforms). The Splice Index algorithm with additional filtering identified much less alternatively spliced genes (3 genes after 0.1 Gy exposure and 17 after 1.0 Gy) (Supplementary Table S3). The FIRMA algorithm results were in keeping with those obtained by the Splice Index (37 genes after 0.1 Gy exposure and 39 after 1.0 Gy) (Supplementary Table S4) with 15 genes being identified as alternatively spliced in response to 1.0 Gy by both algorithms. The same 15 genes were also identified as highly significant by the Partek Alternative Splicing ANOVA algorithm, with 13 of them among the 30 most significant ones according to their p-values (Supplementary Table S2) and, importantly, all 15 genes were differentially expressed (Supplementary Table S1). It has been shown before that radiation-induced alternative splicing occurs predominantly in genes that are differentially expressed at the gene level^11^,^13^.

Although it is not possible to infer the exact sequence identities of specific transcript variants from the gene array results, it was clear that different alternative splicing and transcription mechanisms had been activated in response to radiation exposure. For example, we found evidence of transcription from alternative promoters (e.g. *ASTN2*, *NDUFAF6*, *FDXR* and *PCNA*), alternative transcription initiation (e.g. *ASTN2*), alternative splicing (e.g. *ASTN2* and *FDXR*), and use of alternative 3′-UTRs (e.g. *ASTN2*) (Fig. 3a–d and Supplementary Figure S1). The observed variation in the expression levels between different transcripts was validated by qRT-PCR using transcript-specific primers (Fig. 3). We found significant differences in radiation-induced expression of different variants of *ASTN2* (Fig. 3a), *FDXR* (Fig. 3c) and *PCNA* (Fig. 3d) at 8 h after exposure to 0.1 and 1.0 Gy, while this difference was not significant for *NDUFAF6* after exposure to 1.0 Gy, possibly because of large interindividual variations in the transcriptional response of this gene (Fig. 3b).

Furthermore, several of the probe sets among the 125 genes that were differentially expressed (Supplementary Table S1) have not yet been annotated to a gene. Mapping of their sequences to the mouse genome showed that most of them hybridise to intronic sequences of *PVT1*, *EI24*, *REV3L*, *RNGTT* and *ITPR2* (Supplementary Figure S2). Two other probe sets were found to map to a sequence downstream of *PCNA* and upstream of *REV3L*, respectively. Interestingly, *EI24*, *REV3L*, *ITPR2* and *PCNA* were among the identified radiation-responsive genes (Supplementary Table S1), whereas *Pvt1* was recently identified as a radiation-responsive gene in the embryonic mouse brain^13^. Our data therefore suggest that these probe sets actually identify currently unknown exons of these radiation-responsive genes.

### Radiation exposure induces expression of neighboring genes

The above results indicate that although radiation exposure leads to exon skipping and the use of alternative splice junctions, the mechanism that was most often observed to result in transcript variation was the expression of transcripts from alternative promoters. Since most of these genes are regulated by p53, we hypothesise that the DNA damage response, which is activated after irradiation, induces the expression of p53-dependent transcript variants, as shown previously in both lymphoblastoid cell lines^11^ and the embryonic mouse brain^13^. Interestingly, we also observed that a significant proportion (23 out of 129 annotated genes; 17.8%) of the genes that were differentially expressed after irradiation with 1.0 Gy are genomic neighbors, several of which are transcribed from bidirectional promoters (Supplementary Figure S3). This finding aligns well with a study in which human lung fibroblasts were treated with the p53 activator 5-fluorouracil^16^. Chromatin immunoprecipitation with a p53 antibody followed by next-generation sequencing revealed that about 4% of the high-confidence peaks were located at bidirectional promoters^16^, including some that are identical to those observed by us (e.g. *FAS*-*ACTA2* and *ASTN2*-*TRIM32*).

### Differential expression of distinct exons is more pronounced compared to entire genes

For several genes, individual exons responded much stronger to the irradiation than others. This suggested that signatures of highly responsive exons might be more sensitive and would have greater predictive value as a biomarker of radiation exposure compared to genes, whose expression signals are averaged over the totality of their exons. This observation led us to perform ANOVA at the exon level as well, revealing 706 differentially expressed exons (FDR < 0.05) between different doses of radiation exposure (Supplementary Table S5), with 157 exons being differentially expressed after exposure to both 0.1 and 1.0 Gy (Supplementary Table S6). Comparison of the distributions of fold changes in expression between genes and exons confirmed that the changes in the exon expression levels were more pronounced compared to the genes on a generic basis, especially at the higher dose of 1.0 Gy (Fig. 4a,b). Average fold changes for significant genes and exons after exposure to 0.1 Gy were 1.58 and 1.71, respectively (Fig. 4c), increasing to 1.72 and 2.21 after exposure to 1.0 Gy (Fig. 4d). Clustering of the samples based on the expression levels of the 706 differentially expressed exons using unsupervised hierarchical clustering (Fig. 5a) and PCA (Fig. 5b) resulted in perfect separation of the samples according to radiation dose. Together, these results suggest that exons might be more sensitive radiation biomarkers.

### Prediction analysis of transcriptional markers for radiation exposure

To identify signatures of genes and exons that distinguish between different irradiation doses, we used three supervised classification models: generalised linear models (GLM), Random Forests (RF) and Nearest Shrunken Centroids as implemented by the PAM (Prediction Analysis for Microarrays) algorithm. Additionally, we assessed the suitability of the above-mentioned models for classification of the samples according to exposure dose.

Table 1 shows the results of all models for gene and exon level analysis in the 2-fold cross-validation setting. Both PAM and the RF models attained a very high performance even with a small number of features, and both models outperformed the GLM model. A combined PAM-RF model (features selected by PAM combined with classification by RF) achieved perfect classification with only two gene features (Table 1).

Similar results were obtained for classification at the exon level (Table 1). Here, the PAM and RF models outperformed the GLM model more clearly. Comparison between exon and gene level analysis gave slightly inferior results for exons, with more features being needed to obtain optimal predictive performance. On the other hand, exons performed better than genes when 100 or all features were used. The genes/exons that were selected as the top 20 most important features for each of the classifiers are listed in Table 2. Of these, 12 genes were identified as differentially expressed according to ANOVA and suitable for class prediction by both RF and PAM (*AEN*, *BAX*, *DDB2*, *EDA2R*, *FDXR*, *MDM2*, *POLH*, *RPS27L*, *SESN1*, *TNFRSF10B*, *XPC*, *ZMAT3*).

To evaluate the robustness of gene expression signatures for practical radiation biodosimetry, we tested the predictive performance of our signature on an independent dataset from a study in which whole blood samples from male and female smokers and non-smokers were irradiated with similar doses to those used in our study, i.e., 0.1 Gy, 0.5 Gy and 2.0 Gy^14^. Using ten of our best predictive markers, we were able to classify these independent samples with 97% accuracy (Fig. 6a), which overall is similar to the accuracy obtained in the original publication^14^. Next, we ran the RF model on the dataset of Paul and Amundson, and used the ten best predictors for cross-validation on our samples. This resulted in 100% accuracy (Fig. 6b), i.e., identical to what we found using our 10 best gene predictors (Table 1). Unfortunately, we were not able to independently validate our exon signatures because this dataset did not contain exon-level information.

Furthermore, we compared our results with those of three other studies in which different subjects, radiation doses (up to 8 Gy), dose rates, radiation qualities, time points, cell types and gene expression platforms were used. The specific characteristics of these studies are listed in Supplementary Table S7. Our comparative analysis revealed a very high degree of overlap in radiation-responsive genes between the different experiments, especially between those in which peripheral blood or PBMCs were used (Fig. 6c). Nevertheless, 27 out of 79 genes (34%) that were found to be radiation-responsive in keratinocytes and fibroblasts^17^ were also identified in at least two other studies (Fig. 6c).

Together, these results hint at the existence of a core signature of genes that may be applicable for radiation biodosimetry for a wide range of doses, dose rates, and cell types/specimens after exposure to different radiation qualities.

### Validation of gene and exon expression using qRT-PCR

qRT-PCR was used to validate the expression changes of several identified biomarker genes in PBMCs and whole blood. In general, the majority of the examined genes showed a dose-dependent up-regulation (Pearson’s correlation coefficients ranging between 0.82 and 0.99) after X-irradiation (Fig. 7), although clear differences in the transcriptional response were observed between different genes. For example, most of the tested genes (*ASTN2*, *MDM2*, *NDUFAF6*, *POLH*, *TNFRSF10B*) showed a 2- to 3-fold induction in expression at 8 h after exposure to 1.0 Gy of X-rays, while *DDB2*, *PCNA* and *FDXR* expression levels were 4-, 5- and 25-fold induced, respectively. Furthermore, most of these genes showed significant differences in expression at 8 h after exposure to a dose of 0.1 Gy, demonstrating their sensitivity for this radiation dose at this time point.

To confirm the obtained results, we also assessed the expression levels of these genes at a later time point, i.e. 24 h following irradiation. Our results point to differences in the kinetics of the transcriptional response of these radiation-induced genes: reduced expression levels – but no complete return to basal expression levels - for *FDXR,* similar expression levels for *DDB2*, *MDM2*, *PCNA*, *POLH* and *TNFRSF10B,* and further increased expression levels for *ASTN2* and *NDUFAF6* after 24 h compared to 8 h (Fig. 7). In many cases, however, statistical significance of expression changes after exposure to 0.1 Gy was lost after 24 h.

In addition, we performed a similar qRT-PCR validation experiment using whole blood samples exposed to the same doses. Overall, the obtained results were very similar to those observed in PBMCs (Fig. 7), demonstrating that PBMCs are a suitable model for the transcriptional radiation response of whole blood.

## Discussion

Prompted by the rapid development of high-throughput genomic profiling technologies, several groups have explored the potential of gene expression signatures as biomarkers of (low dose) exposure to ionising radiation^6^,^8^,^18^,^19^,^20^,^21^,^22^. Most of the genes identified in these studies are known to be regulated by p53 and are involved in cell cycle regulation, DNA damage repair and apoptosis and some are already induced after exposure to doses as low as 5–25 mGy^9^,^22^,^23^,^24^. Furthermore, some of these genes allow to discriminate between ionising radiation response profiles and those induced by inflammation^25^. Several genome-wide studies have been undertaken to assess the *in vivo* transcriptional response to ionising radiation using blood samples from radiotherapy patients undergoing either total body irradiation^26^ or local intensity modulated radiotherapy^27^. The results of these investigations indicate that *in vivo* irradiation mainly affects genes involved in pathways that are related to the immune system and inflammatory responses, as well as p53-mediated pathways. Accordingly, induction of p53-dependent genes was observed in patients either undergoing CT scans (up to 4.3 cGy) or receiving (F-18)-fluoro-2-deoxy-d-glucose (0.6 cGy)^24^. Overall, the examined genes were induced in all samples, although differences in the *in vivo* and *in vitro* response were found, especially for doses below 5 cGy^24^. Other studies have identified *in vitro* gene signatures that could accurately predict the *in vivo* radiation exposure status^10^,^28^,^29^. Overall, these studies have shown that the *in vitro* transcriptional radiation response is a reliable model for the *in vivo* situation. Another possibility for biodosimetry studies is the use of animal models. For instance, it was demonstrated that radiation-responsive genes in mice show a response that is similar to that of homologous genes from *ex vivo* human studies^20^,^28^,^30^. On the other hand, gene expression profiles developed through analysis of murine blood radiation responses alone were found to be inaccurate in predicting human radiation exposures^10^.

Unlike the moderate to high radiation doses used in most other studies dealing with transcriptional radiation biomarkers, the X-ray doses applied in this study are low to moderate but nonetheless relevant for medical triage. The moderate dose of 1.0 Gy represents the lower limit of doses that result in acute radiation syndrome^31^ and is associated with a high probability of long-term stochastic health effects. The low dose of 0.1 Gy is not associated with any acute health effects but might require medical follow-up since the risk of long-term effects, particularly cancer, cannot be excluded^32^. To the best of our knowledge, only two studies aimed at identifying a predictive gene signature based on genome-wide data have used doses of 0.1 Gy or below^14^,^22^. However, no cross-validation at the individual donor level was performed in either of these studies, which may have positively biased the results.

One of the initial steps in our study consisted in a gene-level analysis of the microarray data, resulting in a list of genes capable of discriminating between the exposure conditions with high accuracy. A substantial fraction of the radiation-responsive genes were located in close physical proximity on the genome (often as neighbors with bidirectional promoters). We propose that these genes are co-regulated, most likely via activation by p53, or via chromatin loops which can bring promoters in close proximity, thereby exposing them to the same regulatory proteins. This co-regulation may be related to the nature of the stress inflicted on the cells by radiation exposure (i.e. DNA damage) since the frequency of bidirectional promoters is enriched in DNA repair genes compared to other gene classes^33^,^34^. This observation may also be instrumental in identifying currently undiscovered radiation-responsive transcripts. One such new gene we identified as a predictive marker is *PAPPA-AS1* (Table 2), which is a long non-coding RNA transcribed from the opposite strand of *PAPPA*, presumably from a shared bidirectional promoter with *ASTN2*.

The specific microarray platform we used, interrogates the vast majority of exons from multi-exon genes, allowing to analyse the expression data at the exon level as well. Although we could not draw definite conclusions about the exact mechanisms underlying these events, our data are suggestive of the activation of different alternative splicing mechanisms (exon skipping, alternative splice sites, alternative polyadenylation) in response to irradiation. However, the most utilised mechanism appeared to be alternative promoter usage. Importantly, such events result in significant differences in the expression of single exons, while changes in the expression of the gene itself are less pronounced. Forrester and Sprung proposed that dose prediction could be improved by the use of radiation-responsive transcript variants as biomarkers in combination with unresponsive intragenic controls^35^. However, these authors evaluated only three genes, one of which turned out unsuitable for dose prediction^35^.

To the best of our knowledge, comparison of gene and exon signatures for class prediction is a novel approach in biodosimetry, and has only rarely been applied in general biomarker research. Tian *et al.* showed that exons outperformed genes as biomarkers of Tourette syndrome^36^. In another study, gene and exon signatures performed equally well in predicting overall survival in neuroblastoma patients^37^Likewise, our results are indicative of an overall comparable prediction performance of gene and exon signatures.

From our results, and those from other groups^6^,^14^,^18^,^19^,^20^,^21^,^22^, it is now clear that there is a core of approximately 20 genes that can be regarded as robust biomarkers for radiation exposure to a wide range of doses. As such, genome-wide expression studies are undoubtedly highly informative to identify accurate dose-prediction signatures. Nevertheless, using microarrays for mass casualty screening in a radiological emergency situation is not a very realistic approach, due to high costs, limited availability of infrastructures equipped for performing these assays, the rather long response time and the complexity of the analysis. A more cost- and time-efficient alternative would be to use primer- or probe-based assays (such as qRT-PCR) that measure the expression of a limited number of *a priori* identified biomarkers. However, these methods, in contrast to exon-specific microarrays, do not allow to measure the expression of the entire gene but only cover a relatively short region of one or a few exons. Therefore, selection of the most appropriate exons is an imperative prerequisite for using primer- or probe-based assays. We validated the expression profiles of some of the identified genes that were also alternatively spliced in response to irradiation by qRT-PCR using variant- and exon-specific primers for transcripts with different radiation responses, and, for many of the tested genes, we only found a significant difference in expression in low dose-exposed samples with primer pairs amplifying the most sensitive exons. This suggests that these exons may be more sensitive markers for prediction of similar low doses and possibly also those below 0.1 Gy, i.e., doses at which combined exon signals (i.e. gene level) may be no longer predictive. This further highlights the importance of always obtaining prior knowledge about expression levels at the exon level when primer- or probe-based assays are used to perform “gene-level” expression analysis.

Our study has a few limitations. First, only two radiation doses and one time point after irradiation were used to identify the predictive signatures. However, a comparative analysis with previously published studies, as well as validation of the predictive performance of our signatures on an independent dataset containing two additional doses, revealed that our signature also applies to higher doses and longer time points. Second, the gene expression profiles applied in our study stemmed from isolated PBMCs and not from whole blood. To address this, we validated gene expression using qRT-PCR on *ex vivo* irradiated blood samples, revealing highly similar transcriptional responses to radiation in PBMCs and whole blood for the investigated genes.

In conclusion, we have shown that gene and exon signatures are equally performing in predicting exposure to radiation doses within the 0.1–1.0 Gy range at 8 h after exposure. We have generated a robust fingerprint for predictive biodosimetry and especially triage of individual radiation casualties. Implementation of a dedicated assay based on the identified biodosimetric panel may lead to improved point-of-care diagnostics for radiological accidents. Finally, we have shown the importance of evaluating gene expression at the level of single exons for transcriptional biomarker discovery in general.

## Methods

**Figure 1:**
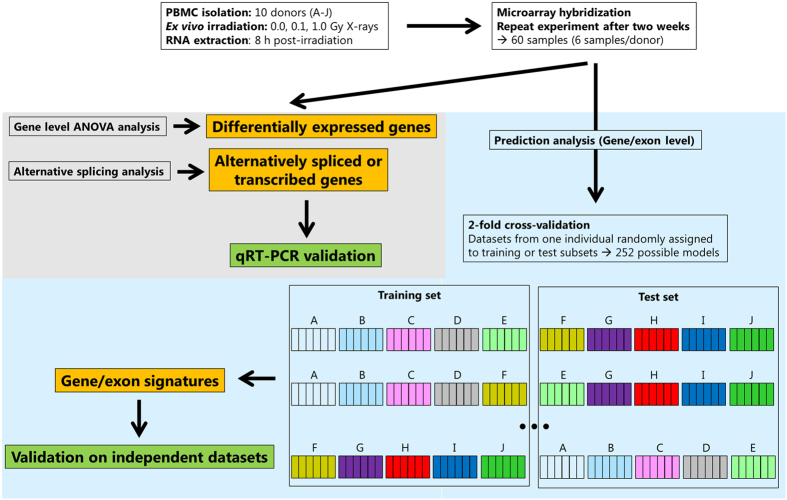
Outline of the strategy to identify gene and exon signatures suitable for prediction of exposure to doses of 0.1 and 1.0 Gy of X-rays. Isolated PBMCs from 10 healthy donors were irradiated and RNA was extracted 8 h after irradiation. RNA was hybridised onto Affymetrix Human Gene 1.0 ST arrays and differentially expressed and alternatively spliced genes were identified and validated using qRT-PCR. Three classification algorithms were employed to identify predictive gene and exon signatures and their predictive performance was tested using 2-fold cross-validation (boxes labeled with letters A–J represent all the samples from each out of ten donors (0.0, 0.1 and 1.0 Gy, in duplicates). The best performing gene signature as identified by the RF model was further evaluated on an independent, publicly available dataset.

**Figure 2:**
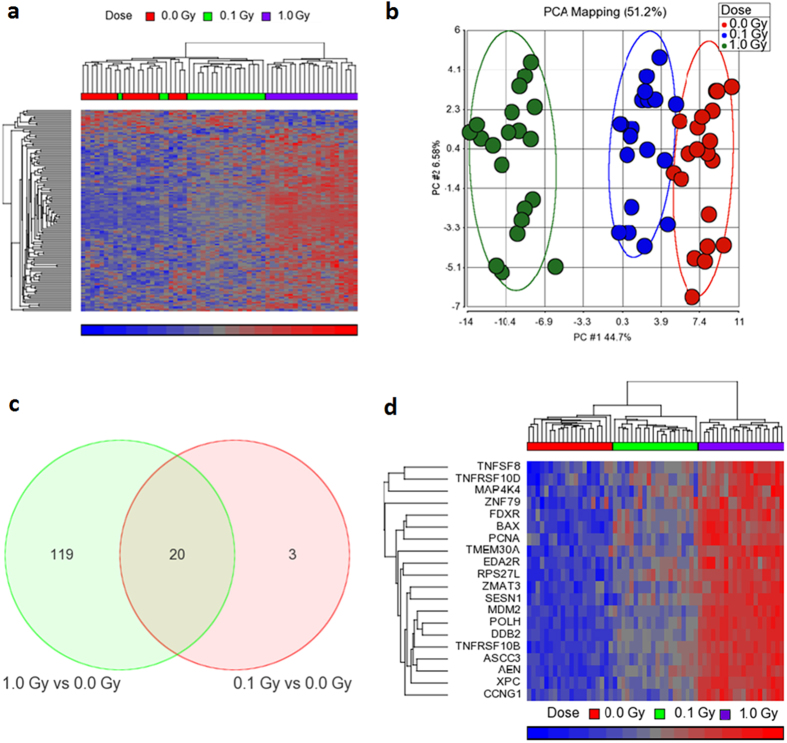
Gene expression changes in PBMCs in response to irradiation at 8 h after exposure. In total, 125 genes were identified as differentially expressed by ANOVA (FDR corrected *p*-values < 0.05) between 0.1 Gy and 1.0 Gy samples and sham-irradiated controls. (**a**) Unsupervised hierarchical clustering analysis of these 125 genes (rows) showed good separation of samples (columns) depending on the dose of exposure. Red: high gene expression, blue: low gene expression. (**b**) 2D Principal Components Analysis (PCA) also separated samples depending on the dose. Each circle represents the expression profile of the 125 significantly differentially expressed genes in one sample. The percentage of the variance explained by the first and the second principal components is 44.7% and 6.58%, respectively. Ellipses represent two standard deviations. (**c**) Venn diagram showing the overlap of differentially expressed genes after exposure to 0.1 and 1.0 Gy of X-rays. (**d**) Unsupervised hierarchical clustering analysis of the 20 overlapping genes showed perfect separation of samples depending on the dose of exposure. Red: high gene expression, blue: low gene expression.

**Figure 3:**
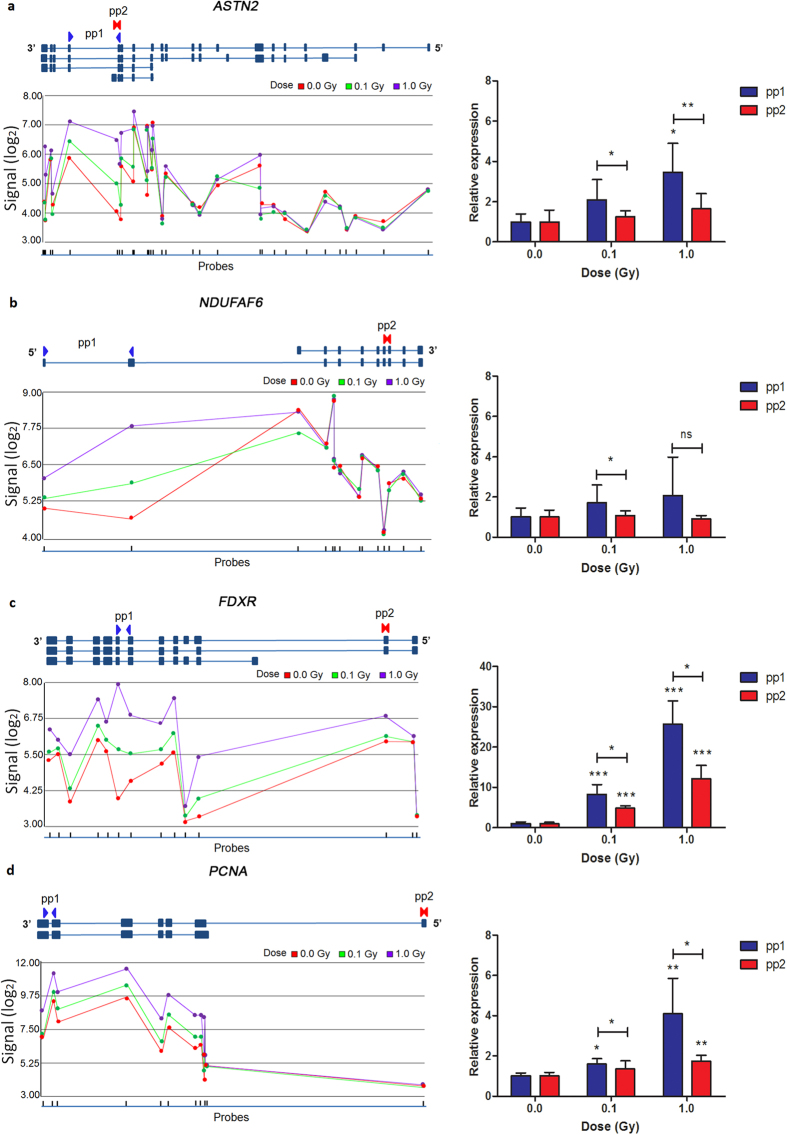
Radiation-induced alternative splicing. (**a–d**). Upper panels show genomic organisation of a few transcript variants from the UCSC database of *ASTN2* (**a**), *NDUFAF6* (**b**), *FDXR* (**c**) and *PCNA* (**d**). Each blue box represents an exon and the interconnecting lines represent introns. Lower panels show the log_2_ normalised intensity signals for each microarray probe, located on each specific exon shown above (error bars were left out to increase clarity). Arrows indicate the location of the primer pairs (pp) used for qRT-PCR validation. qRT-PCR validation results for each primer pair are shown on the right. Graphs represent mean + standard deviation. Statistical comparison was performed using the paired *t*-test (*p-value < 0.05, **p-value < 0.005, ***p-value < 0.0001, ns: not significant).

**Figure 4:**
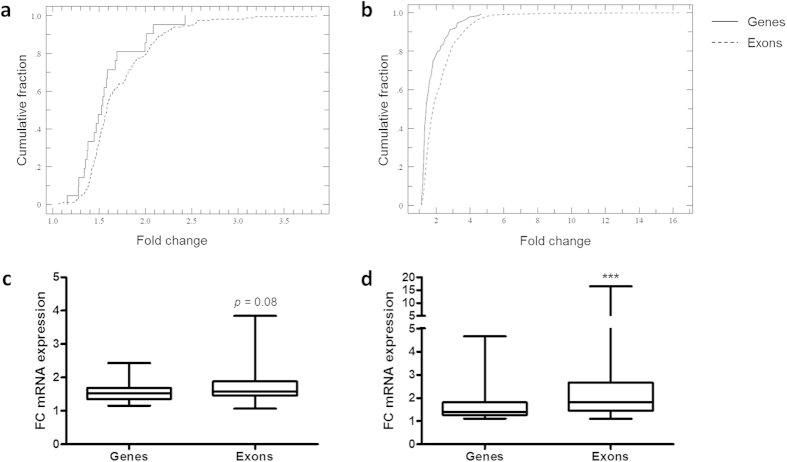
Fold-change induction of exon expression is more pronounced compared to gene expression. (**a,b)** Cumulative distributions of fold changes in expression for significantly differentially expressed genes and exons after exposure to 0.1 Gy (**a**) and 1.0 Gy (**b**) of X-rays. *p*-values for the difference between distributions for genes and exons according to the Kolmogorov-Smirnov test were 0.195 and 0.000 for 0.1 Gy and 1.0 Gy, respectively. (**c,d)** Box plots depicting fold changes in expression for the same data as in (**a**,**b**). (**c**) Genes and exons upregulated at 0.1 Gy. (**d**) Genes and exons upregulated at 1.0 Gy. Centerlines show the median, boxes represent the range between the first and third quartiles and whiskers represent the highest and lowest values. ***p-value < 0.0001 (Two-tailed Mann-Whitney test).

**Figure 5:**
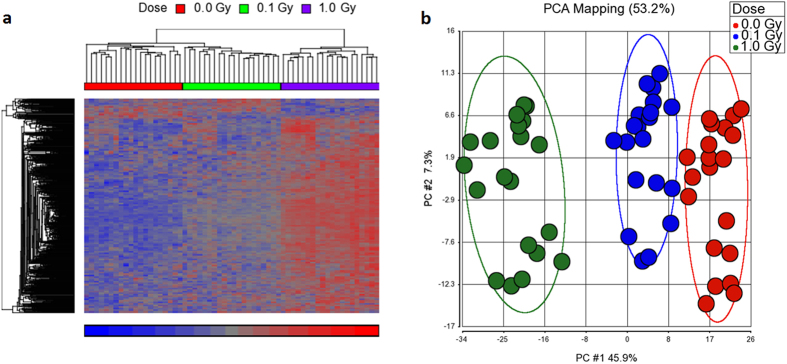
Probe (exon) expression changes in PBMCs in response to irradiation. In total, 706 exons were identified as differentially expressed by ANOVA (FDR corrected *p*-values < 0.05) between different irradiation doses. (**a)** Unsupervised hierarchical clustering analysis of the 706 exons (rows) showed perfect separation of samples (columns) depending on the dose of exposure. Red: high gene expression, blue: low gene expression. (**b)** 2D Principal Components Analysis (PCA) also separated samples depending on the dose. Each circle represents the expression profile of the 706 significantly differentially expressed exons in one sample. The percentage of the variance explained by the first and the second principal components is 45.9% and 7.3%, respectively. Ellipses represent two standard deviations.

**Figure 6:**
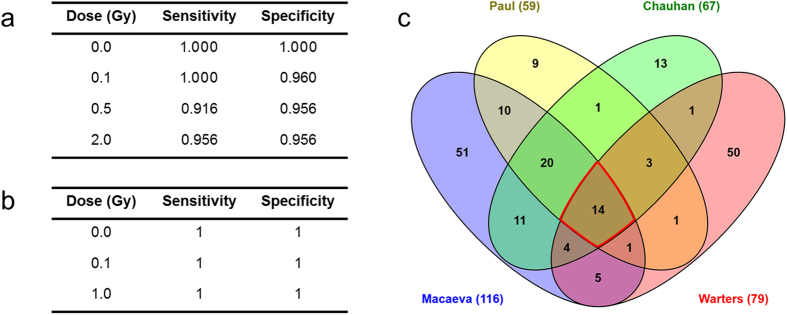
Gene signatures are robust predictive biomarkers of exposure to radiation. **(a)** Five-fold cross-validation of our 10 best predictive genes on the dataset of Paul and Amundson^14^ resulted in 97% accuracy for sample classification. (**b)** Five-fold cross-validation of the 10 best predictive genes from the dataset of Paul and Amundson^14^ as identified by the Random Forests model resulted in 100% accuracy for classification of our samples. (**c)** Overlap between differentially expressed genes from this study and gene signatures identified by Paul and Amundson^6^, Chauhan and co-authors^18^ and Warters and co-authors^17^. Common genes between all datasets are *DDB2*, *POLH*, *MDM2*, *RPS27L*, *FDXR*, *CCNG1*, *TRIAP1*, *SESN1*, *FBXO22*, *PPM1D*, *ANKRA2*, *CDKN1A*, *TRIM22*, and *BBC3*.

**Figure 7:**
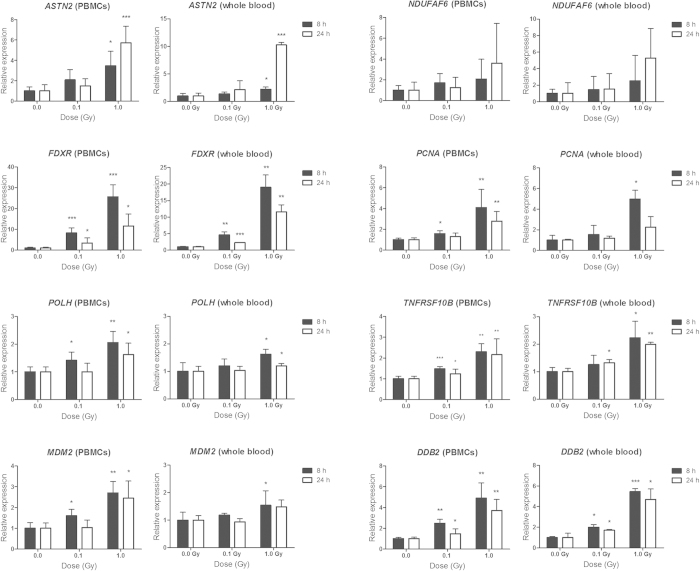
qRT-PCR validation of the microarray results. qRT-PCR results for *ASTN2, NDUFAF6, FDXR, PCNA*, *POLH*, *TNFRSF10B*, *MDM2* and *DDB2* genes at 8 and 24 h after irradiation of PBMCs and whole blood. Graphs represent mean + standard deviation. Statistical comparison was performed using the paired *t*-test (*p-value < 0.05, **p-value < 0.005, ***p-value < 0.0001).

Experimental procedures are schematically summarised in Fig. 1.

### Blood collection and PBMCs isolation

Peripheral blood samples used for microarrays were collected from 10 healthy, non-smoking Caucasian donors (5 males/5 females; age range: 23–50 years; median age: 28 years) in EDTA vacutainer tubes. All procedures followed were approved by the local SCK•CEN Ethics Committee and were carried out in accordance with the ethical standards of the Helsinki Declaration of 1975, as revised in 2000. All donors had signed an informed consent form prior to blood donation. Within 30–60 min of blood drawing, PBMCs were isolated by centrifugation on Histopaque-1077 (Sigma-Aldrich, Bornem, Belgium) density gradient according to the manufacturer’s instructions. Isolated cells were suspended at a density of 10^6^ cells/ml in LGM-3 culture medium (Lonza, Walkersville, MD, USA) and were allowed to equilibrate to culture conditions at 37 °C in a humidified 5% CO_2_ atmosphere. Two weeks later, the experiment was repeated using fresh PBMCs from the same donors, resulting in a total of 60 samples for microarray hybridisation. For quantitative RT-PCR (qRT-PCR) validation, blood collected from 5 different donors (1 male and 4 females), from whom informed consent had been obtained, was subjected to identical procedures as the samples used for microarray hybridisation, unless otherwise indicated. To confirm the results obtained for isolated PBMCs in whole blood samples, blood was collected from 3 donors (1 male and 2 females), from whom informed consent had been obtained, in EDTA vacutainer tubes, which were then directly used for irradiation.

### *In vitro* irradiation

Cells were irradiated “free-in-air” at 21 °C in a horizontal position with single doses of 0.1 and 1.0 Gy of X-rays from a Pantak HF420 RX generator at an air kerma (K_air_) rate of 0.26 Gy/min or were sham-irradiated. More detailed information on the irradiation setup can be found in the Supplementary Methods. Following *in vitro* irradiation, PBMCs were incubated at 37 °C in a humidified 5% CO_2_ atmosphere. Whole blood samples were incubated on a rocking platform at 37 °C without additional CO_2_ supply for the indicated time points.

### RNA extraction

RNA from irradiated and sham-irradiated PBMC samples was extracted 8 h after irradiation for microarray hybridisation and 8 and 24 h for qRT-PCR validation. RNA from whole blood samples used for qRT-PCR validation was extracted 8 and 24 h after irradiation. For RNA isolation from PBMCs, a combined approach consisting of the TRIzol® reagent (Invitrogen, Carlsbad, CA, USA) extraction method and purification on Qiagen RNeasy columns (Qiagen, Venlo, The Netherlands), was used. More detailed information on the RNA extraction procedure can be found in the Supplementary Methods. The QIAamp RNA Blood Mini Kit (Qiagen, Venlo, The Netherlands) was used to extract RNA from whole blood samples. The starting quantity of blood was 1.5 ml per sample. All procedures were performed following the manufacturer’s instructions. RNA concentration was measured on a NanoDrop-2000 spectrophotometer (Thermo Scientific, Erembodegem, Belgium) and the quality of total RNA samples was assessed using Agilent 2100 Bioanalyser (Agilent Technologies, Santa Clara, CA, USA). All samples had a RNA Integrity Number >8 and were therefore considered as suitable for further processing for microarrays and qRT-PCR.

### Microarray hybridisation

Gene expression profiling was performed using the GeneChip® Human Gene 1.0 ST Array (Affymetrix, Santa Clara, CA, USA), which interrogates 28,536 well-annotated genes with 253,002 distinct probe sets, allowing expression analysis at both gene and exon level. Since each probe corresponds to one exon in most of the cases, we refer to probe set-level analysis as exon-level analysis. More detailed information on the microarray hybridisation procedure can be found in the Supplementary Methods. All microarray data are available in MIAME compliant format at the ArrayExpress database (www.ebi.ac.uk/arrayexpress) under the accession number E-MTAB-3463.

### Microarray data analysis

The obtained microarray data were imported into Partek Genomics Suite, version 6.6 (Partek Inc., St Louis, MO, USA) as .CEL-files. Probe summarisation and probe set normalisation were done using the Robust Multichip Analysis (RMA) algorithm^38^, which includes background correction, quantile normalisation and log_2_ transformation. Microarray data were analysed both at the level of probe sets and probe sets summarised to genes using a three-way ANOVA with dose, gender and batch as factors. Inclusion of batch in the model allowed correcting for differences between experiments resulting from different scanning days of the microarrays. To correct for multiple testing, we used the false discovery rate (FDR) as described by Benjamini and Hochberg^39^ to adjust *p*-values (FDR < 0.05). We also performed linear contrasts between two specific groups (0.1 Gy vs control and 1.0 Gy vs control) within the context of ANOVA. The coefficients of the levels in the two compared groups add up to 0. The computations of *p*-values are based on Least-squares means, which are the means adjusted by other factors. Genes and exons were considered significantly differentially expressed between the two groups if adjusted *p*-values were < 0.05 with no fold change cutoff. Pearson’s correlation coefficient was used to assess dose dependence of the gene expression levels. We used the Principal Components Analysis tool of the Partek software as an exploratory method to detect groupings in the dataset as well as to spot possible outliers. This technique is used to describe the structure of high dimensional data by reducing its dimensionality. It is a linear transformation that converts *n* original variables (genes or exons, in our case) into *n* new variables, which have three important properties: principal components are ordered by the amount of variance explained, they are uncorrelated and they explain all variation in the data. PCA was performed at both gene and exon level using normalised expression values. The correlation method applied to calculate the dispersion matrix adjusted the data to be standardised to a mean of 0 and standard deviation of 1.

### Alternative splicing analysis

To predict alternative splicing in irradiated samples compared to controls, we used three different methodologies, since it is known that alternative splicing analysis from gene arrays is prone to generate false positive results^40^. First, we performed Alternative Splicing ANOVA in Partek. A FDR-corrected *p*-value of < 0.05 was considered significant for alternative splicing events. To further reduce the number of false positives, we excluded the probe sets with log_2_ value < 3.0 (noise level) in all samples from analysis, except for the cases where there was a significant difference in expression of a single exon between the groups (*p* < 0.05). Next, we used two supplementary methods to perform a pairwise comparison of the samples (0.1 Gy vs 0.0 Gy and 1.0 Gy vs 0.0 Gy) to further increase the reliability of our results. Gene Array Analyzer^41^ is an on-line tool that uses the Splice Index algorithm^42^ and allows the user to perform more advanced filtering, i.e., removing probe sets that are not expressed in at least one group, removing genes (transcript clusters) that are not expressed in both groups, discarding probe sets with high potential for cross-hybridisation and those with very large gene-level normalised intensities. Software parameters were set to default values, except for the Splice Index cutoff, which was set to 0.5. AltAnalyze^43^ is an open-source software utilising the FIRMA algorithm which is another method for detection of alternative splicing^44^. Software parameters were set to default values, except for the Minimum alternative exon score and the Maximum absolute gene-expression change, which were set to 0.5 and 50, respectively.

### Positional Gene Enrichment analysis (PGE)

The PGE tool^45^ is available at http://homes.esat.kuleuven.be/~biouser/pge/. We used default parameters to detect positional enrichment of radiation-responsive genes.

### Prediction analysis

The following statistical models were evaluated with regard to their predictive performance and identification of a minimal list of genes and exons capable of discriminating between exposure conditions: GLM, RF^46^ and PAM method^47^. A more detailed description of these models can be found in the Supplementary Methods.

To compare the predictive performance of genes and exons, two versions of the dataset were constructed: (a) a version measuring expression changes at the gene level, and (b) a version measuring expression changes at the exon level.

Cross-validation was used to assess whether classification models could be constructed to predict the different conditions. The original dataset was split into a part for model training (training set) and a part for model evaluation (test set), where both sets are disjoint. In our case, the cross-validation had to be executed at the level of individuals, since otherwise correlations between different conditions of the same biological sample might have led to overoptimistic results. This setting best mimics the true setup where new, unseen biological samples need to be classified by the model. A higher number of possible train-test combinations results in a more robust assessment of model performance, since higher numbers of models could be averaged. Therefore, we finally used 2-fold cross-validation for the prediction analyses (Fig. 1).

For model hyperparameters that needed to be tuned (such as the lambda value for GLM or the threshold for PAM), an internal cross-validation on the training partition in each cross-validation loop was used. This optimal value was subsequently used to train a final model on the training partition in each cross-validation loop, and produce results for the test partition in each cross-validation loop.

Performance of the individual models was evaluated by calculating the AUC in which a value of 0.5 corresponds to random prediction behavior and a value of 1 to optimal prediction performance. This is known to be a robust estimator of model performance over different model decision thresholds.

To validate our results on an independent publicly available dataset, we retrieved data from Paul and Amundson^14^ (GEO accession number GSE23515), describing a set of 95 samples from 24 individuals of different age, gender and smoking status exposed to different doses of radiation (0.0, 0.1, 0.5 and 2.0 Gy). After pre-processing, probe sets that did not map to gene symbols and probe sets containing more than 25% empty values were filtered out. After this filtering step, 23,031 probe sets were kept for further analysis. Subsequently, feature importance rankings were derived from classifiers basedon RF and PAM as described above. The overlap between the top 100 genes from our study and those from Paul and Amundson was higher based on the RF ranking; therefore, the cross-validation was performed using this model.

The Venny on-line tool^48^ was used to compare gene lists and create Venn diagrams: http://bioinfogp.cnb.csic.es/tools/venny/index.html

### Reverse transcription and qRT-PCR

The following genes were selected for qRT-PCR validation: *DDB2*, *POLH*, *MDM2*, *TNFRSF10B*, *FDXR, ASTN2*, *NDUFAF6*, and *PCNA*. RNA samples from 5 donors were used for cDNA synthesis using the GoScript™ Reverse Transcription System (Promega, Leiden, The Netherlands) with random hexamer primers. For each gene, qRT-PCR reactions were run in duplicate using the MESA GREEN® qRT-PCR kit (Eurogentec, Seraing, Belgium) on an Applied Biosystems® 7500 Real-Time PCR instrument following the manufacturer’s instructions. To determine the efficiency and specificity of the designed primers, we ran a standard curve experiment with melt curve for every primer pair. Primer sequences and reaction efficiencies are listed in Supplementary Table S8. qRT-PCR data were analysed by 7500 Software v2.0.6 and Microsoft Excel using the Pfaffl method^49^. The relative amount of transcript of the selected genes was normalised to *PGK1* and *HPRT1* using the geometric mean of these reference genes^50^. Relative expression levels were tested for statistical significance using the paired *t*-test; *p*-values of <0.05 were considered significant. Pearson’s correlation coefficient was used to assess dose dependence of the gene expression levels.

## Additional Information

**How to cite this article**: Macaeva, E. *et al.* Radiation-induced alternative transcription and splicing events and their applicability to practical biodosimetry. *Sci. Rep.* **6**, 19251; doi: 10.1038/srep19251 (2016).

## Supplementary Material

Supplementary Information

Supplementary table S1

Supplementary table S2

Supplementary table S3

Supplementary table S4

Supplementary table S5

Supplementary table S6

## Figures and Tables

**Table 1:** Predictive performance at gene (G) and exon (E) levels using 2, 5, 10, 20, 50, 100 or all features as measured by the AUC.

Model	2		5		10		20		50		100		ALL	
	G	E	G	E	G	E	G	E	G	E	G	E	G	E
**GLM Net**	0.985	0.942**	0.965	0.926**	0.932	0.922**	0.949	0.946	0.953	0.952	0.952	0.951**	0.959	0.953
**RF**	0.985	0.968**	0.998	0.993**	1.000	0.998**	1.000	1.000	1.000	1.000	1.000	1.000**	0.918	0.955
**PAM**	0.999	0.997	1.000	1.000	1.000	1.000	1.000	1.000	1.000	1.000	0.997	1.000**	0.999	1.000**
**PAM + RF**	1.000	0.997	1.000	0.999	1.000	1.000	1.000	1.000	1.000	0.999**	1.000	1.000	0.917	0.953

GLM: Generalized Linear Models; RF: Random Forests; PAM: Nearest Shrunken Centroids.

^**^p < 0.01 for comparison between gene and exon level (Mann-Whitney U-test).

**Table 2:** Top 20 most important features selected by PAM and Random Forests models for the data at gene and exon level.

	Random Forests		PAM	
	Genes	Exons	Genes	Exons
1.	**DDB2**	ASCC3 exon 43 (8128473)	**DDB2**	DDB2 exon 4 (7939743)
2.	**POLH**	**DDB2 exon 5 (7939744)**	**POLH**	**DDB2 exon 5 (7939744)**
3.	**AEN**	XPC exon 15 (8085487)	**MDM2**	FDXR exon 8 (8018242)
4.	**TNFRSF10B**	**TNFRSF10B exon 9 (8149735)**	**TNFRSF10B**	DDB2 exon 8 (7939747)
5.	**RPS27L**	RPS27L exon 1 (7989499)	**RPS27L**	POLH exon 5 (8119864)
6.	**FDXR**	PCNA exon 2 (8064851)	**AEN**	AEN exon 3 (7985775)
7.	**MDM2**	XPC exon 2 (8085501)	PCNA	TNFRSF10B exon 9 (8149737)
8.	**ZMAT3**	ASCC3 exon 41 (8128476)	**XPC**	**POLH exon 11 (8119870)**
9.	**XPC**	ASCC3 exon 6 (8128513)	ASCC3	DDB2 exon 3 (7939742)
10.	**C12orf5**	**POLH exon 11 (8119871)**	**ZMAT3**	RPS27L exon 3 (7989497)
11.	**EDA2R**	ASCC3 exon 14 (8128504)	**GADD45A**	MDM2 exon 4 (7956994)
12.	NUP133	**POLH exon 10 (8119869)**	**SESN1**	DDB2 exon 9 (7939748)
13.	FBXO22	MDM2 exon 10 (7957001)	**C12orf5**	POLH exon 4 (8119863)
14.	**GADD45A**	POLH exon 1 (8119860)	**EDA2R**	MDM2 exon 7 (7956997)
15.	ZNF79	RPS27L exon 2 (7989498)	**FDXR**	DDB2 exon 6 (7939745)
16.	**BAX**	TNFRSF10B exon 7 (8149739)	CCNG1	**POLH exon 10 (8119869)**
17.	**SESN1**	ASCC3 exon 13 (8128505)	**BAX**	PCNA exon 3 (8064850)
18.	ACTA2	TNFRSF10B exon 5 (8149756)	PPM1D	**TNFRSF10B exon 9 (8149735)**
19.	ITGA2	PHPT1 exon 2 (8159444)	FAS	NDUFAF6 exon 2 (8147426)
20.	TMEM30A	ASCC3 exon 37 (8128481)	PAPPA-AS1	DDB2 exon 7 (7939746)

Features are listed according to their internal weight (importance) in every model. The numbers in brackets in the Exons columns indicate corresponding Affymetrix Probe set IDs. Overlapping genes and exons are indicated in bold.
