# Supplementary material for: Radiation-induced alternative transcription and splicing events and their applicability to practical biodosimetry
Source: Sci Rep. 2016 Jan 14;6:19251. doi: 10.1038/srep19251 (PMC4725928; doi:10.1038/srep19251)
Supplement: Supplementary Information [file srep19251-s1.pdf]

# Radiation-induced alternative transcription and splicing events and their applicability to practical biodosimetry

Ellina Macaeva[1, 2], Yvan Saeys[3, 4], Kevin Tabury[1], Ann Janssen[1], Arlette Michaux[1], Mohammed A. Benotmane[1], Winnok H. De Vos[2, 5], Sarah Baatout[1, 2], Roel Quintens[1]*

[1]Radiobiology Unit, Belgian Nuclear Research Centre, SCK•CEN, Mol, Belgium
[2]Department of Molecular Biotechnology, Ghent University, Ghent, Belgium
[3]Data Mining and Modelling for Biomedicine Group, VIB Inflammation Research Center, Zwijnaarde, Belgium
[4]Department of Respiratory Medicine, Ghent University, Ghent, Belgium
[5]Department of Veterinary Sciences, University of Antwerp, Antwerp, Belgium
*Corresponding author: roel.quintens@sckcen.be

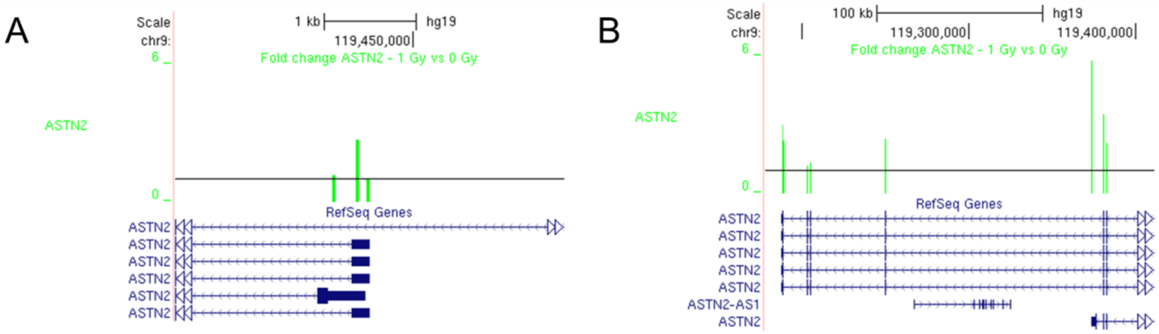


**Figure S1. Possible alternative splicing events responsible for differences in expression signals for different probes of the *ASTN2* gene.** (A) Radiation-induced expression of a truncated first exon of the short ASTN2 variant. (B) Radiation-induced splicing of two cassette exons and expression of an alternative 3’ exon. In both panels top tracks indicate fold changes for individual probes (green lines) in 1.0 Gy samples compared to 0.0 Gy. The vertical line indicates no change. Bottom track shows different transcript variants. Arrows indicate the 5’ to 3’ orientation of the gene.

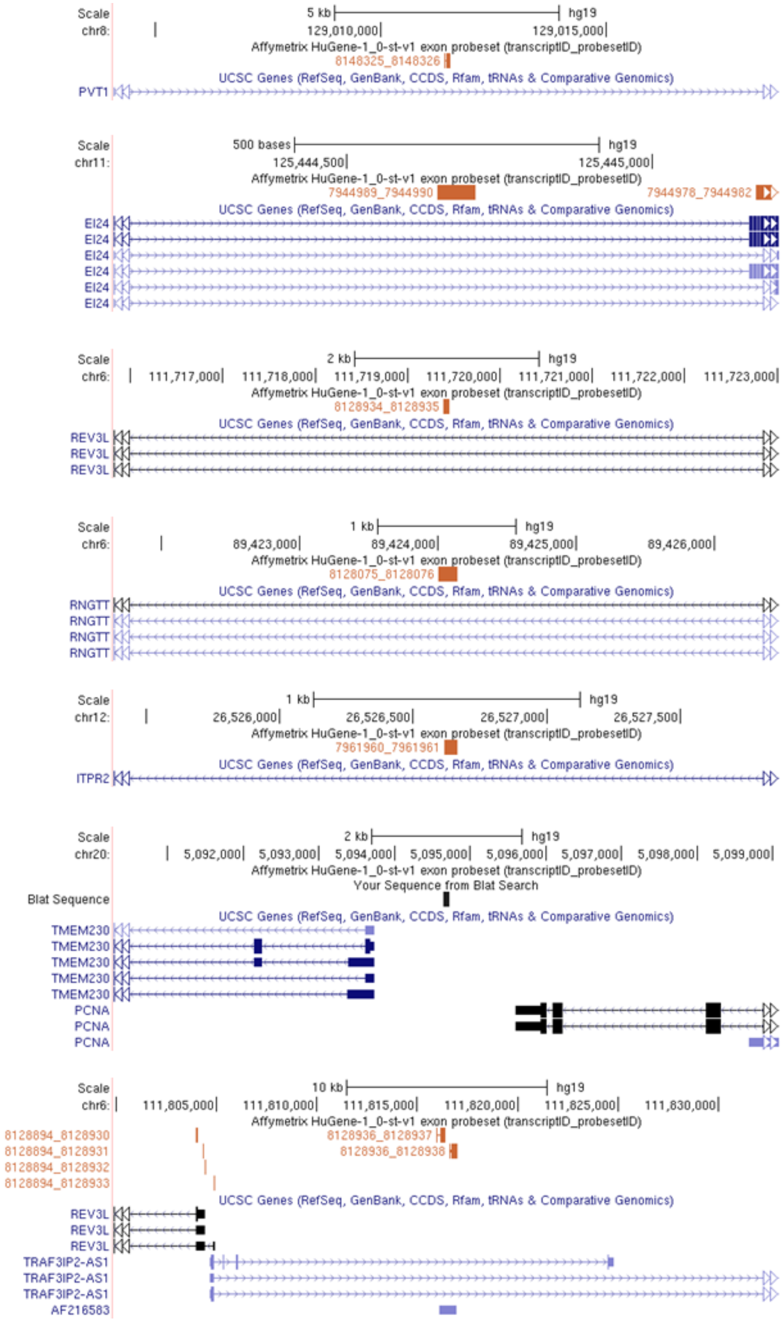


**Figure S2.** Mapping of the probesets currently not annotated to a gene from Table S1. In each panel, top tracks indicate Affymetrix probesets while bottom tracks indicate known splice variants. Arrows indicate 5' to 3' orientation of the gene.

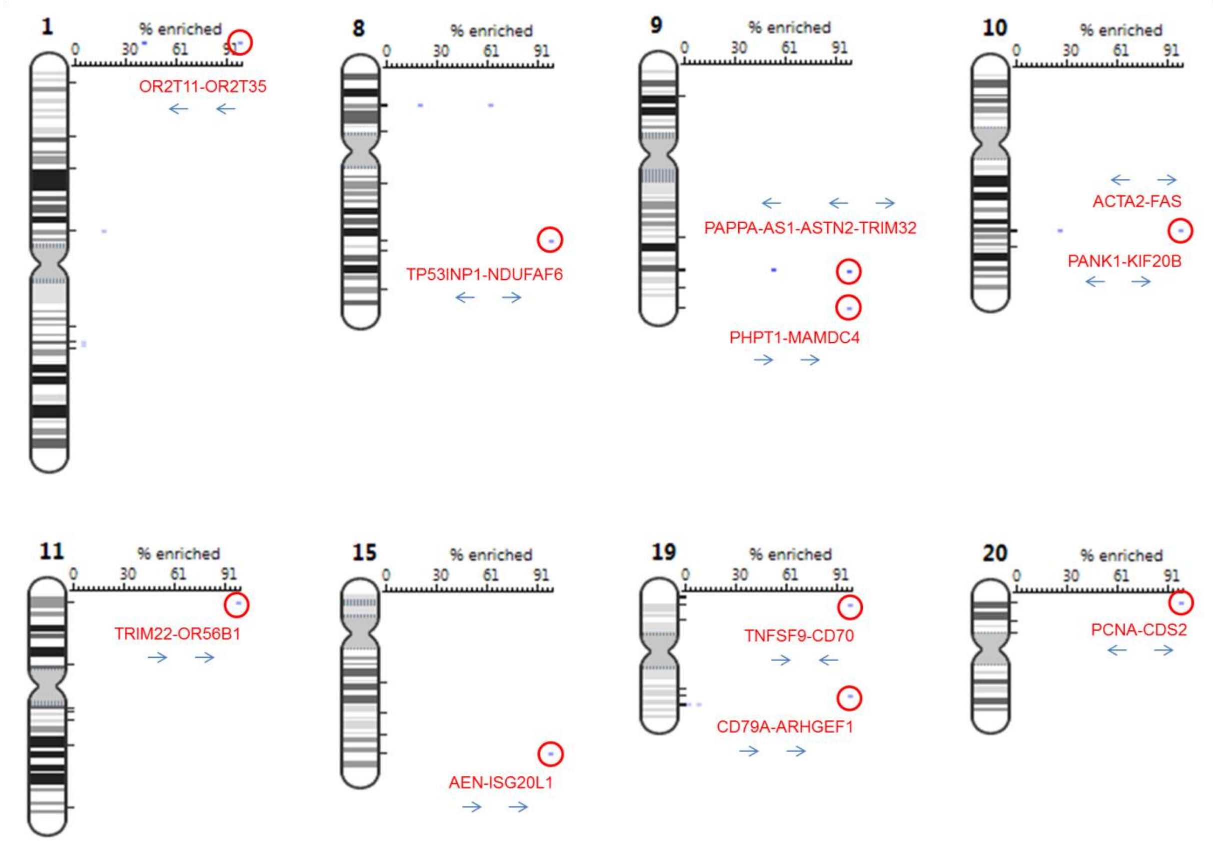


**Figure S3. Positional gene enrichment analysis shows significant co-localisation of radiation-responsive genes.** Scale bar indicates percentage of enrichment with 100% enrichment corresponding to genomic neighbors. Arrows indicate the 5’ to 3’ orientation of the genes. Please note that the separate clusters *ACTA2-FAS* and *PANK1-KIF20B* are in close proximity (< 1 Mb) on chromosome 10.

| Table S7: Characteristics of studies used for comparison analysis | | | | | | |
|---|---|---|---|---|---|---|
| Reference | Cell type | Radiation quality | Radiation dose (Gy) | Dose rate | Time points | Array platform |
| This study | PBMCs | X-rays | 0; 0.1; 1.0 | 0.26 Gy/min | 8 h | Affymetrix Human Gene 1.0 ST array |
| Paul and Amundson[1] | Peripheral blood | γ-rays | 0; 0.5; 2; 5; 8 | 0.82 Gy/min | 6 h, 24 h | Agilent Whole Human Genome Oligo Microarrays |
| Chauhan et al.[2] | PBMCs | Alpha particles | 0; 0.5; 1.0; 1.5 | 0.98 Gy/h | 24 h | Illumina human-12 v2 RNA BeadChips |
| Warters et al.[3] | Keratinocytes, fibroblasts | γ-rays | 0; 0.1; 1; 5 | 0.47 Gy/min | 4 h | Agilent 44K arrays |


1. Paul, S. & Amundson, S. A. Development of gene expression signatures for practical radiation biodosimetry. *International journal of radiation oncology, biology, physics* **71**, 1236-1244, doi:10.1016/j.ijrobp.2008.03.043 (2008).

2. Chauhan, V., Howland, M. & Wilkins, R. Identification of gene-based responses in human blood cells exposed to alpha particle radiation. *BMC medical genomics* **7**, 43, doi:10.1186/1755-8794-7-43 (2014).

3. Warters, R. L., Packard, A. T., Kramer, G. F., Gaffney, D. K. & Moos, P. J. Differential gene expression in primary human skin keratinocytes and fibroblasts in response to ionizing radiation. *Radiation research* **172**, 82-95, doi:10.1667/RR1677.1 (2009).

**Table S8. Primer sequences for qRT-PCR**

| Gene | Forward primer (5' → 3') | Reverse primer (5' → 3') | Reaction efficiency (%) |
|---|---|---|---|
| *ASTN2* (pp1) | TCTGCAGCATAAGAAAGTGGATGA | CAATCAAGTTGGCTTCATCCCTG | 95 |
| *ASTN2* (pp2) | TCTGCAGCATAAGAAAGTGGATGA | GACCAGCTGCTACACAAGATGT | 103 |
| *DDB2* | TGAACATGGACGGCAAAGA | TGGCCAGGAACCAATCAC | 93 |
| *FDXR* (pp1) | GCAAGTGGCCTTCACCATTAAG | CCTTGATCTTGTCCTGGAGACC | 88 |
| *FDXR* (pp2) | GCTTCTGCCACCATTTCTCC | CTTTAGCAGGTGTTGGGCC | 104 |
| *HPRT1* | TCAGGCAGTATAATCCAAAGATGGT | AGTCTGGCTTATATCCAACACTTCG | 96 |
| *MDM2* | CGATTATATGATGAGAAGCAACAAC | CTCTTTCACAGAGAAGCTTGG | 88 |
| *NDUFAF6* (pp1) | AAAGAGAGACTGGAGCCACCT | GAGGAATGTGAAATGCTGATTGGC | 94 |
| *NDUFAF6* (pp2) | GCAACACCATATCATGGGAGCA | CTTGGTTCCTCCGTAGAAAGTCC | 97 |
| *PCNA* (pp1) | GCACTGAGGTACCTGAACTT | TCTTCATCCTCGATCTTGGG | 109 |
| *PCNA* (pp2) | CCCTGGTTCTGGAGGTAAC | CAGGTCAGCAAGCATTTGTC | 104 |
| *PGK1* | CAAGAAGTATGCTGAGGCTGTCA | CAAATACCCCCACAGGACCAT | 102 |
| *POLH* | GGGAAGCCAGTGTTGAAGT | CTTGTACAGCACTGGTCAGAT | 94 |
| *TNFRSF10B* | GTGGATGGAACATCCTGTAACT | AGTACGCACAAACGGAATGA | 93 |

## Supplementary Methods

### *In vitro* irradiation

The beam quality can be approximated to H-250 (ISO4037): 250 kV, 15 mA, 1.2 mm Al equivalent inherent filtration and 1 mm Cu additional filtration. The $K_{air}$ at the reference position was measured using a NE2571 ionisation chamber (SN309) connected to a Farmer 2500 electrometer. The chamber, together with the electrometer, was calibrated in terms of $K_{air}$ and the traceability to the international standards was assured. The reference point of the ionisation chamber was placed at the same distance with the reference position of the samples. The ionisation chamber was always placed in the beam, next to the samples, for a precise measurement of the time integrated $K_{air}$. The stability of the X-ray generator during the irradiation was verified in this way.

### RNA extraction

For RNA isolation from PBMCs a combination of the TRIzol® reagent (Invitrogen, Carlsbad, CA, USA) extraction method and the purification on Qiagen RNeasy columns (Qiagen, Venlo, The Netherlands) was used. Briefly, $5x10^6$ cells were lysed in 1 ml of TRIzol® reagent and further processed following the manufacturer's recommendations. Following the RNA precipitation with isopropanol, the obtained pellet was resuspended in 1 ml of ethanol and transferred to the RNeasy column. Further purification was done according to the manufacturer's instructions.

### Microarray hybridisation

Ten µg of cRNA, synthesised and purified from 0.25 µg of total RNA using the Ambion® WT Expression kit (Ambion, USA) was used for cDNA synthesis, followed by cDNA fragmentation and labeling with the GeneChip® Terminal Labeling kit (Affymetrix, Santa Clara, CA, USA).

Fragmented and labeled cDNA was hybridised to Human Gene 1.0 ST arrays (Affymetrix, Santa Clara, CA, USA) using the GeneChip® Hybridization, Wash and Stain kit (Affymetrix, Santa Clara, CA, USA) (hybridization module) and hybridization controls (Affymetrix, Santa Clara, CA, USA) with rotation at 45°C for 16 hours. After hybridization, arrays were washed and stained using GeneChip® Hybridization, Wash and Stain kit (stain module) after which the arrays were immediately scanned using an Affymetrix GeneChip® Scanner.

**Predictive analysis**

Generalized linear models were trained using the R glmnet package. These methods build a regularised linear model which uses the lasso penalty to perform feature selection, resulting in only relevant features to receive nonzero weights. A multinomial model was used to model the three-class classification problem, and an internal five-fold cross-validation was used to tune the model's internal parameter lambda. Feature importance measures were then derived from the weights of the linear model. The Random Forest based classification model uses an ensemble of randomised decision trees to perform classification. We used a collection of 1000 decision trees to build these models, and subsequently used the internal feature importance mechanism based on entropy reduction to obtain feature importance values. The Nearest Shrunken Centroid Classifier gradually shrinks the average gene expression centroids of the two groups to the overall centroid. The non-differentially expressed genes are removed first as the distance between the centroids of two groups is small in this case and the group centroids of these genes will therefore quickly reach the overall centroid. Differentially expressed genes, in contrast, will “survive” the shrinkage much longer and will have a higher probability of being used for classification. The optimal level of shrinkage is determined with ten-fold cross-validation, which is used to select the

number of genes for class prediction. Finally, the centroids of these genes are used to classify the new samples to the nearest centroid.

While each of these classifiers has an internal mechanism to select informative features based on their internal weight or importance, we also experimented with explicitly reducing the number of features describing the data. To this end, internal model information was used to weigh features and keep only the most important ones. We explored the following number of features: 2, 5, 10, 20, 50, 100 most important features, and finally also the traditional setting where all features were used. To perform feature selection in an unbiased way, we again only selected the most important features from the training partition in each cross-validation loop. Subsequently, a model with only the selected number of features was retrained on the full training partition, and executed on the test partition within each cross-validation loop.
